# Impact of latency‐reversing agents on human macrophage physiology

**DOI:** 10.1002/iid3.590

**Published:** 2022-12-08

**Authors:** Laurent Hany, Marc‐Olivier Turmel, Corinne Barat, Michel Ouellet, Michel J. Tremblay

**Affiliations:** ^1^ Axe des Maladies Infectieuses et Immunitaires, Centre de Recherche du Centre Hospitalier Universitaire de Québec‐Université Laval Québec Canada; ^2^ Département de Microbiologie‐Infectiologie et Immunologie, Faculté de médecine Université Laval Québec Canada

**Keywords:** human animals, immunodeficiency diseases, monocytes/macrophages cells, viral/retroviral infections

## Abstract

**Introduction:**

HIV‐1 eradication is hindered by the presence of inducible long‐lived reservoirs of latently infected cells which rapidly disseminate viral particles upon treatment interruption. Eliminating these reservoirs by the so‐called shock and kill strategy represents a crucial concept toward an HIV‐1 cure. Several molecules called latency‐reversing agents (LRAs) are under intensive investigations to reactivate virus gene expression. These studies are mainly conducted on CD4^+^ T cells where LRAs are well tolerated and did not induce global cellular activation. However, despite their broad spectrum, the putative impact of LRAs on other cellular reservoirs such as macrophages is still ill‐defined.

**Methods:**

We investigated the impact of the protein kinase C (PKC) activator bryostatin‐1, bromodomain inhibitor JQ1 and histone deacetylase inhibitor romidepsin used either alone or in combination on human primary monocyte‐derived macrophages (MDMs).

**Results:**

We demonstrate that bryostatin‐1, JQ1, and romidepsin or their combinations are not toxic at nanomolar concentrations but induce metabolic and morphologic alterations of MDMs. Bryostatin‐1 triggered the secretion of pro‐inflammatory cytokines, while JQ‐1 decreased it. Phagocytosis and endocytosis were modestly impaired upon bryostatin‐1 treatment whereas efferocytosis was markedly downregulated by romidepsin. Despite its pro‐inflammatory profile, bryostatin‐1 did not induce classically activated macrophage markers. Finally, we reveal that conditioned medium from bryostatin‐1‐treated macrophages did not potentiate its reactivation feature.

**Conclusions:**

Our study reveals that LRAs can diversely impact basic physiologic features of human primary macrophages and could potentially decrease reactivation of nearby CD4^+^ T cells latently infected with HIV‐1. Our observations further stress the need to include different cell populations when assessing HIV‐1 cure strategies.

## Graphical abstract

Latency‐reversing agents affect metabolism and morphology of macrophages.

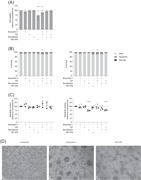

## 1 INTRODUCTION

With viremic rebound occurring after decades of antiretroviral therapies, it became obvious that HIV‐1 persistence could not be alleviated by standards treatments.^1^ Identification of latently infected cells as the primary mechanism for HIV‐1 persistence paved the way to the development of therapies aimed at reversing latency. This concept theorized by Deeks nearly a decade ago relies on latent provirus reactivation to purge out HIV‐1 reservoirs by the host immune system combined with an antiviral regimen to prevent new cellular infections.^2^

This strategy involves the use of latency‐reversing agents (LRAs) to shock latent cells by triggering HIV‐1 production.^3^ Since latency establishment was primarily identified and vastly studied in CD4^+^ T cells, reversing strategies were similarly massively investigated in this cell type. Thus, numerous studies revealed that HIV‐1 could be reactivated in CD4^+^ T cells with different potencies using several agents belonging to different classes of LRAs primarily acting on epigenetic mechanisms such as histone deacetylase inhibitors (HDACis) and histone methyltransferase inhibitors (HMTis), and on the recruitment or activation of transcription factors, such as protein kinase C activators (PKCas) and bromodomain, and extra‐terminal domain inhibitors (BETis).^3^, ^4^, ^5^ A better reactivation was achieved using combination of LRAs classes with PKCas/HDACis and PKCas/BETis resulting in the best outcome.^5^, ^6^ More importantly, LRAs treatment did not induce cell death or global T‐cell activation as revealed by surface markers and cytokine secretion, a necessary condition to avoid clonal expansion or cellular toxicity.^5^, ^6^, ^7^, ^8^, ^9^ However, because LRAs are almost exclusively studied on CD4^+^ T cells, their impact on other cell subsets such as macrophages remains widely overlooked. The rational of investigating this latter population reside in the fact that (i) these cells are considered as stable reservoirs and highly suspected to harbor HIV‐1 latency,^10^, ^11^ (ii) they are more resistant to HIV‐1‐induced cytotoxicity and immune clearing mechanisms,^12^, ^13^ (iii) they are less permissive to antiretroviral treatments,^14^, ^15^, ^16^ and (iv) they are involved in innate and acquired immune responses.

This latter feature is of prime interest when assessing the safety and potency of LRAs treatments. It has been shown that LRAs did not modulate neither cellular activation nor cytokine secretion in CD4^+^ T cells.^5^ An increase in cell death and exhaustion markers as well as an impaired clearance of HIV‐1‐infected CD4^+^ T cells has been reported after treatment of CD8+ T cells with bryostatin‐1 and romidepsin^17^, ^18^ although a clinical study found no evidence of T‐cell dysregulation after romidepsin infusions.^4^ Moreover, some LRAs could also either decrease or increase the antiviral activity of natural killer cells.^19^, ^20^ Finally, our group previously demonstrated that bryostatin‐1 treatment increased the secretion of pro‐inflammatory cytokines by astrocytes accompanied with neutrophils invasion and NETosis while JQ1 had opposite effects.^21^ Moreover, both JQ1 and bryostatin‐1 downregulated the yeast phagocytic capacity of this cell type.^22^ Thus, innate and acquired immune responses of various cell populations can be altered by LRAs.

The aim of this study was to investigate LRAs impact on macrophages viability and primary innate immune functions. To do so, one agent of each of the three most studied classes of LRAs namely romidepsin (HDACi), bryostatin‐1 (PKCa), and JQ1 (BETi) were used either alone or in combination in human monocyte‐derived macrophages (MDMs). Our results revealed that while some LRAs could affect profoundly cellular morphology, they did not alter cell viability. Bryostatin‐1 treatment rapidly increased the expression of pro‐inflammatory cytokines while JQ1 reduced it. LRAs had a modest impact on phagocytosis and micropinocytosis, however, bryostatin‐1 and romidepsin strongly impaired transferrin uptake and efferocytosis of apoptotic T cells, respectively. These marked alterations were accompanied by a modulation of some polarization markers by romidepsin and bryostatin‐1 without clear relation to the classical M1 and M2 dichotomy. Finally, we demonstrate that conditioned medium from bryostatin‐1‐treated MDMs had no effect on the reactivation of latently HIV‐1‐infected J‐Lat cells.

## 2 MATERIALS AND METHODS

### 2.1 Ethics statement and cell culture

This study was approved by the Bioethics Committee from the Centre Hospitalier Universitaire de Québec‐Université Laval. Peripheral blood samples were collected from healthy donors respecting guidelines of the Institutional Bioethics Committee with written consent provided by all participants. Experiments were conducted in accordance with the Institutional guidelines and regulations.

Peripheral blood mononuclear cells were collected after Ficoll‐Hypaque (Corning Life Science) gradient centrifugation and seeded for 2 h at 37°C to allow adherence of monocytes. Cells were then washed extensively with Dulbecco's phosphate‐buffered saline (DPBS) (Corning Life Science) to remove nonadherent cells. Monocytes were maintained for 3 d in RPMI 1640 culture medium (Corning Life Science) supplemented with 10% (v/v) of AB human serum (Valley Biomedical), penicillin/streptomycin (Gibco, Thermofisher Scientific) thereafter referred as complete culture medium and 25 ng/ml of macrophage colony‐stimulating factor (GenScript). Cells were washed extensively in DPBS and maintained for 3 additional days in complete culture medium to obtain nonpolarized MDMs. Cells were then washed and incubated for 1 h at 37°C with AccutaseTM (Invitrogen, Thermofisher Scientific) and detached by gentle scrapping. MDMs were seeded at various cell concentrations usually 1.5–2 × 10^5^ for a minimum of 24 h before further processing either in Ultra‐Low Attachment plates (Corning) for experiments requiring flow cytometry studies or in regular tissue culture‐treated plates (Corning) when cell detachment was not necessary.

The reporter cell line HEK‐Blue™ TNF‐α (Invivogen) was maintained in Dulbecco's modified Eagle's culture medium (Invitrogen) supplemented with 10% (v/v) heat inactivated Fetal Bovine Serum (FBS) (Corning Life Science) for the first two passages and supplemented with 100 µg/ml of Zeocin™ and 1 µg/ml of puromycin for cell maintenance, both from Invivogen.

The following reagents were obtained through the NIH HIV Reagent Program, Division of AIDS, NIAID, NIH: J‐Lat Full Length Cells (clone 10.6), ARP‐9849 and J‐Lat Tat‐GFP Cells (clone A2), ARP‐9854, contributed by Dr. Eric Verdin and maintained in RPMI 1640 culture medium supplemented with 10% (v/v) heat inactivated FBS and antibiotics at 37°C.

### 2.2 Antibodies and reagents

Bryostatin‐1 (used at 20 nM) (Sigma‐Aldrich), JQ1 (500 nM), and Romidepsin (5 nM) (Cayman Chemicals) were used either alone or in various combinations. A combination of TNF‐α (20 ng/ml) and IFN‐γ (10 ng/ml) both from BioLegend was used as positive control to induce a pro‐inflammatory like phenotype also referred as M1.

Mouse anti‐human ICAM‐1 (CD54) (12‐0549‐42), fixable viability dye eFluor™ 450 (65‐0863‐18), 780 (65‐0865‐18), pHrodo™ Green Zymosan (P35365), pHrodo™ Green *Escherichia coli* BioParticles™ (P35366), pHrodo™ Green Dextran 10,000 MW (P35368), Vybrant® DiD Cell‐Labeling Solution and trypsin 2.5% were all purchased from ThermoFisher Scientific.

Annexin V‐CF Blue 7‐AAD Apoptosis Staining/Detection Kit (ab214663) was purchased from Abcam (Cambridge, United Kingdom). QUANTI‐Blue™ (rep‐qb1) was obtainned from Invivogen. Phase fluid endocytosis inhibitor chlorpromazine and micropinocytosis inhibitor 5‐(N‐Ethyl‐N‐isopropyl) amiloride thereafter called EIPA were purchased from Sigma‐Aldrich.

Apotracker Green, CCL2, CCL5, IL‐10, and IL‐8 ELISA were purchased from Biolegend.

### 2.3 Phagocytosis assay

MDMs were seeded in complete culture medium in ultralow adherence plates and treated with LRAs for 24 h. Culture medium was then removed and a solution of complete culture medium supplemented with 25 µg/ml of pHrodo (zymosan or *E. coli*) was added and incubated at 37°C for 20–25 min. Cells were then gently washed twice in ice cold phosphate buffered saline (PBS) to stop phagocytosis. Trypsin was then added for 5 min incubation at 37°C to remove bounded but not ingested particles. Trypsin reaction was stopped by adding ice cold complete culture medium. Cells were detached after a 30 min incubation in PBS supplemented with 5 mM EDTA (PBS/EDTA) at 4°C followed by gentle ups and downs and washes. Cells were finally stained with fixable viability dye, washed several times in ice cold PBS/EDTA before their processing by flow cytometry.

### 2.4 Endocytosis assay

MDMs were seeded in complete culture medium in ultralow plates and treated with LRAs for 24 h. For studying clathrin‐mediated endocytosis, MDMs were detached, stained with viability dye for 30 min at 4°C, washed and preincubated in warm RPMI supplemented with 1% BSA medium for 30 min at 37°C to remove transferrin from human serum origin. Chlorpromazine (10 µM) was added in selected wells. Cells were then washed extensively and incubated with 25 µg/ml of Alexa 633‐labeled transferrin at 37°C or at 4°C in RPMI + 1% BSA for 30 min. Cells were washed, fixed in 2% formaldehyde and analyzed by flow cytometry. For phase fluid endocytosis, MDMs were incubated with warm culture medium supplemented with 25 µg/ml of pHrodo Green Dextran 10,000 MW in the presence or not of EIPA (25 or 50 µM) for 35 to 40 min at 37°C. Cells were washed extensively with ice cold PBS and detached at 4°C in PBS/EDTA for 30 min. MDMs were finally stained with viability dye, washed and analyzed by flow cytometry.

### 2.5 Efferocytosis assay

For the preparation of apoptotic cells, Jurkat cells were resuspended at a concentration of 1 × 10^6^/ml in RPMI 1640 supplemented with 10% (v/v) heat inactivated fetal bovine serum (FBS) (Corning Life Science), antibiotics, and 1 µM of staurosporin and cultured overnight. Cells were then washed to remove staurosporin and incubated at 5 × 10^6^ per 100 µl in 800 nM of Apotracker Green in DPBS for 20 min at room temperature. Cells were finally washed twice to remove remaining fluorescent dye before co‐incubation.

MDMs were seeded in complete culture medium in ultralow attachment plates and stained with Vybrant® DiD (5 µl per ml) for 30 min at 37°C. Cells were washed twice and treated with LRAs for 24 h. Culture medium was then replaced by a solution of complete culture medium containing stained apoptotic Jurkat cells at a 1:4 ratio (MDMs: Jurkat cells). Apoptotic cells were spun down and co‐incubated at 37°C for 30–40 min. Cells were then gently washed twice in ice cold PBS to stop efferocytosis and incubated for 30 min at 4°C in PBS/EDTA. After gentle ups and downs to detach cells and several washes in ice cold PBS/EDTA, cells were fixed in 2% formaldehyde for 30 min at 4°C and analyzed by flow cytometry. The efferocytosis efficacy was calculated as the ratio of DiD‐Apotracker green double positive cells over total DiD positive cells.

### 2.6 Viability, apoptosis, and metabolic assays

MDMs were seeded in complete culture medium and treated with LRAs for 6 or 24 h. Metabolic activity was quantified using CellTiter 96 AQueous nonradioactive cell proliferation assay following the manufacturer's instructions (Promega). For viability and apoptosis determination, cells were detached by incubation in PBS/EDTA for 30 min at 37°C. MDMs were stained with a viability dye (viability assay) for 30 min at 4°C or with a solution of 7AAD and annexin V (apoptosis assay) according to the manufacturer's instructions. Cells were then washed extensively and analyzed by flow cytometry.

### 2.7 J‐Lat reactivation test

J‐Lat cells clone 10.6 and A2 were seeded in a 96 well plate at 10^5^ cells per well for 24 h. Half culture medium volume was replaced with conditioned medium from 24 h LRA‐treated MDMs or similarly treated LRA containing medium. After another 24 h of cell culture, J‐Lat cells were analyzed by flow cytometry where HIV‐1 reactivation was quantified based on GFP expression.

### 2.8 Gene expression and cytokine secretion

MDMs were treated with LRAs for 6 or 24 h after which conditioned medium was collected and centrifugated to separate supernatant containing cytokines from non‐adherent cells. Supernatants were used for cytokine detection using Human CCL2, CCL5, IL‐8, and IL‐10 ELISA or the reporter cell line HEK‐Blue™ TNF‐α according to manufacturer instructions. For ICAM‐1 membrane detection, cells were treated for 24 h, detached and processed by flow cytometry. Total RNA from adherent cells was extracted following instructions of Macherey‐Nagel's NucleoSpin® RNA kit (Duren, Germany). Purified RNA was reverse‐transcribed into cDNA using Moloney Murine Leukemia Virus reverse transcriptase (Promega), random primers (Roche), and dNTP mix (Thermofisher Scientific). Gene expression of CCL2, CCL5, IL‐8, IL‐10, TNF‐α, IDO‐1, CD206, and TGM2 was achieved using oligonucleotides listed in Table 1 and PowerUp SYBR green master mix (Applied Biosystems) on a Quanstudio 6 Flex system apparatus (Applied Biosystems). Gene expression was normalized based on 18S RNA expression using the 2^‐ΔΔ^ct method.^23^

**Table 1:** list of primers sequences from Integrated DNA Technologies used in this study

Target	Forward sequence 5’–3’	Reverse sequence 5’–3’
CCL2	CCCCAGTCACCTGCTGTTAT	TGGAATCCTGAACCCACTTC
CCL5	CTGCTTTGCCTACATTGCCC	TCGGGTGACAAAGACGACTG
TNF‐α	CCTGCTGCACTTTGGAGTGA	GAGGGTTTGCTACAACATGGG
IL‐8	TAGCAAAATTGAGGCCAAGG	AAACCAAGGCACAGTGGAAC
IL‐10	GAACCAAGACCCAGACATCAA	CATGGCTTTGTAGATGCCTTTC
CD206	AGATATGCCAGGGCGAAAGC	GGTGGGTTACTCCTTCTGCC
IDO‐1	TGGCCAGCTTCGAGAAAGAG	TGGCAAGACCTTACGGACATC
TGM2	TGTGGCACCAAGTACCTGCTCA	GCACCTTGATGAGGTTGGACTC
18S	TAGAGGGACAAGTGGCGTTC	CGCTGAGCCAGTCAGTGT

### 2.9 Macrophages polarization

MDMs polarization was achieved by stimulating for 24 h unpolarized MDMs or M0 with either TNF‐α (2 ng/ml) and IFN‐γ (20ng/ml) or LPS (1 µg/ml) and IFN‐γ (20 ng/ml) to generate classically activated or pro‐inflammatory macrophages, referred as M1 and IL‐4 (100 ng/ml) to generate alternatively or anti‐inflammatory macrophages, referred as M2. Total RNA was extracted, reverse‐transcribed into cDNA and gene expression of targeted genes was quantified by qPCR.

### 2.10 Flow cytometry analysis

For antibody labeling, cells were blocked for 30 min at 4°C with blocking buffer (PBS containing 5 mM EDTA, 1% BSA, 20% Normal Goat Serum and 10% AB‐Human Serum) and stained in the same buffer for cell surface protein for an additional 15 min at 4°C. Finally, cells were fixed in a 2% formaldehyde solution for 30 min at 4°C, washed and resuspended in PBS/EDTA before their acquisition on BD FACSCelesta (BD Biosciences) apparatus. Data were analyzed on FlowJo software version 10 for Windows.

### 2.11 Statistical analysis

All statistical tests were performed using GraphPad Prism, version 9.03. Test description and number of independent donors (*n*) portrayed as symbols are indicated in the figure legends. Despite matching symbols, donors from different experiments may be unrelated. Statistics of raw data collected as percentages were calculated on their logit transformed values, whereas cytokine secretion values were Log‐transformed to achieve a normal distribution. Normality was assessed using the Shapiro‐Wilk normality test. Data compared to the untreated condition were considered statistically significant for *p*‐values ≤ 0.05.

## 3 RESULTS

### 3.1 Treatment of MDMs with LRAs induced significant morphological and metabolic changes

**Figure 1:**
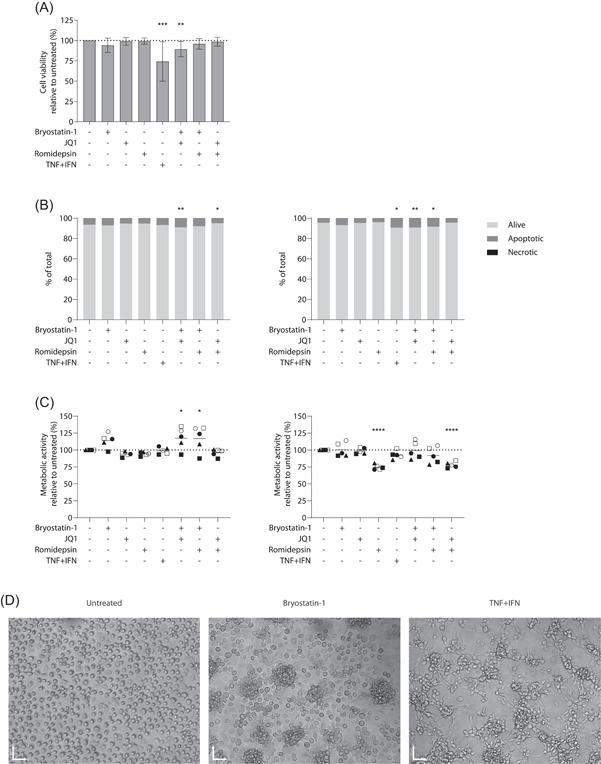
Latency‐reversing agents (LRAs) affect metabolism and morphology of monocyte‐derived macrophage (MDMs). MDMs were first treated with LRAs or TNF + IFN for 6 or 24 h. (A) Cells were stained with an exclusion dye to assess cell viability at 24 h. Results are depicted as percentage of positive cells over the untreated condition (*n* = 11). (B) Percentage of apoptotic and necrotic cells labeled with Annexin V/7AAD at 6 h (left panel) and 24 h (right panel) of treatment (*n* = 5). (C) Metabolic activity was assessed after 6 (left panel) or 24 h (right panel) using a MTS assay (*n* = 5). Data are represented as the metabolic activity relative to untreated cells. (D) MDMs seeded in ultralow adherence plates were treated with bryostatin‐1 or TNF + IFN and visualized by inverted microscope with magnification ×10 after 24 h of treatment. One representative donor is shown, *X* and *Y* scales: 70 µm. (A) Freidman test with Dunn's multiple comparison, (B,C) one‐way ANOVA with Dunnett's multiple‐comparison test (*p ≤ .05. **p ≤ .01. ***p ≤ .005. ****p ≤ .001)

In this study, LRAs were used at concentrations sufficient to reverse HIV‐1 latency in CD4^+^ T cells without triggering cell activation and toxicity. Treatment with tumor necrosis factor‐alpha (TNF‐α) and interferon gamma (IFNγ) was used as a pro‐inflammatory stimulus control. We first tested the toxicity of the studied LRAs on human MDMs. To this end, we assessed cell viability, apoptosis and necrosis after 6 or 24 h of treatment with LRAs using flow cytometry. None of the LRAs tested when used alone could modulate cell viability at 24 h (Figure 1A). Only combinations of TNF‐α and IFNγ (TNF + IFN) and bryostatin‐1 with JQ1 appeared to significantly decrease viability of MDMs with a mild effect for the LRAs (90% viability of the untreated condition). We next assessed the effect of LRAs on cellular apoptosis and necrosis after 6 or 24 h of treatment. While necrotic cells were hardly detected in all conditions, apoptosis was slightly inhibited after 6 h of treatment with JQ1 and romidepsin while TNF + IFN and bryostatin‐1 combinations induced a mild increase (Figure 1B, left panel). This pattern was amplified after 24 h of treatment and confirmed the low toxicity of LRAs in MDMs (Figure 1B, right panel).

Next, we monitored the metabolic activity of MDMs following treatment with LRAs using a colorimetric MTS assay. Bryostatin‐1 treatment for 6 h increased cell metabolic activity (Figure 1C, left panel), which is consistent with the observed rapid acidification of the cell culture medium. After 24 h however, bryostatin‐1‐treated cells metabolism returned to untreated levels whereas romidepsin induced a 25% decrease (Figure 1C, right panel), an effect paralleled by a reduced acidification of the culture medium. These results tend to imply that bryostatin‐1 increases rapidly the metabolic activity of macrophages while romidepsin decreases it over time. These opposite effects may explain the intermediate metabolic activity pattern observed with LRAs combinations.

Finally, when observed in microscopy, macrophages seeded in ultralow adherence plates displayed an organization into cell clumps when treated with bryostatin‐1, a morphological alteration resembling the pro‐inflammatory “like” phenotype induced by TNF + IFN (Figure 1D). No such features were observed following JQ1 or romidepsin treatment. Altogether, our data thus suggest that the concentrations of LRAs used in these in vitro experiments are not toxic for macrophages but can alter their overall morphology and metabolic activity.

### 3.2 Treatment with LRAs alters the expression and secretion of specific cytokines in MDMs

**Figure 2:**
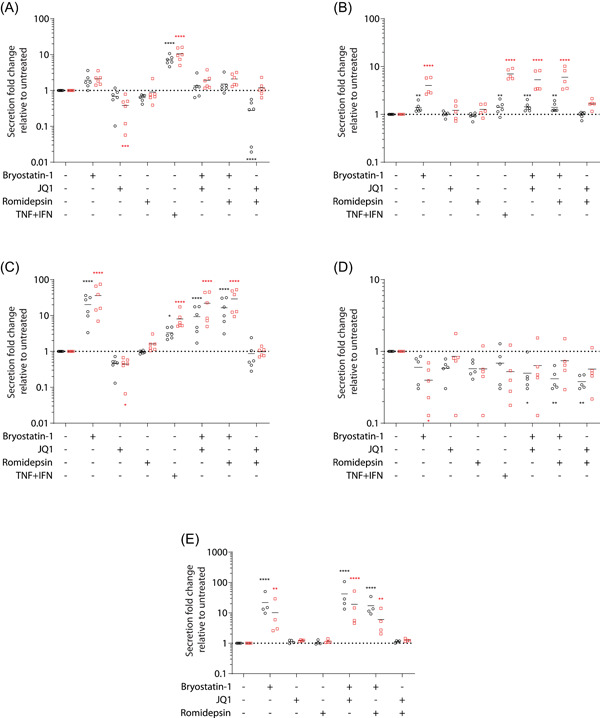
Modulation of cytokine production by latency‐reversing agent (LRA) treatment. monocyte‐derived macrophage (MDMs) were either left untreated or treated with LRAs for 6 (○) or 24 h (**□**). Supernatant was collected and assayed for detection of CCL2 (A), CCL5 (B), IL‐8 (C), IL‐10 (D), and TNF‐α (E) by ELISA or reporter cell line. Results from 4 to 6 donors are represented as secretion fold change over untreated condition. Statistics were performed on log‐transformed of raw data. Due to low level of detection, a nonparametric test (Friedman with Dunn's multiple comparison) was done for IL‐10 while one‐way ANOVA with Dunnett's multiple‐comparison test was performed for the other listed cytokines (*p ≤ .05. **p ≤ .01. ***p ≤ .005. ****p ≤ .001)

**Figure 3:**
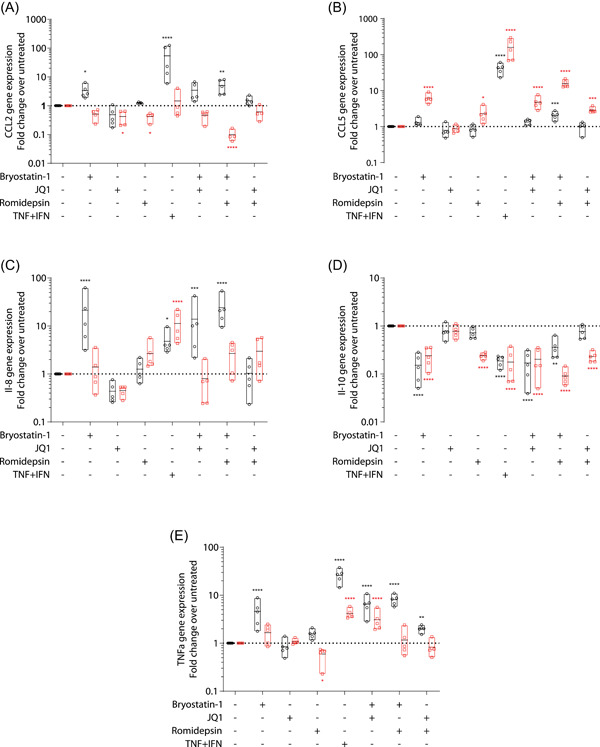
Monocyte‐derived macrophages (MDMs) were either left untreated or treated with latency‐reversing agents (LRAs) for 6 (○) or 24 h (□). mRNAs were then purified and quantified by RT‐qPCR for CCL2 (A), CCL5 (B), IL‐8 (C), IL‐10 (D), and TNF‐α (E) gene expression. Results from 4 to 5 donors were normalized on 18S RNA expression and represented as fold change over untreated condition. Horizontal solid lines displayed maximum, mean, and minimum values for each condition. One‐way ANOVA with Dunnett's multiple‐comparison test (*p ≤ .05. **p ≤ .01. ***p ≤ .005. ****p ≤ .001)

Since MDMs morphology and metabolism is affected by bryostatin‐1 and based on our previous observations indicating that some LRAs enhanced production of pro‐inflammatory cytokines in astrocytes,^21^ we assessed the LRAs‐mediated effect on several cytokine secretion (Figure 2) and gene expression levels (Figure 3) in MDMs. To this end, cells were treated with LRAs for 6 or 24 h after which secretion and gene expression of CCL2 (panel A), CCL5 (panel B), IL‐8 (panel C), IL‐10 (panel D), and TNF‐α (panel E) were monitored by quantitative reverse transcription PCR (RT‐qPCR), commercial ELISA or reporter cell line as described in the material and methods section. Results show that bryostatin‐1 treatment rapidly increased the secretion of pro‐inflammatory cytokines (Figure 2), namely two‐fold for CCL2 and more than ten‐fold for IL‐8 and TNF‐α and induced a lower increase for CCL5, while decreasing the anti‐inflammatory cytokine IL‐10 (two‐fold at 6 h). These secretions were either maintained (CCL2), increased (IL‐8 and CCL5) or decreased (TNF‐α and IL‐10) by a longer treatment period (i.e., 24 h). In contrast, JQ1 treatment caused a minor decrease of CCL2 and IL‐8 secretion over time without a major effect on the other tested cytokines. Finally, romidepsin treatment did not substantially modulate cytokine secretion except for a two‐fold decrease in IL‐10. We did not detect any modulation of IL‐6 and IFNα/β secretion after LRA treatment (data not shown). Moreover, combinatory regimens did not produce potent synergic or antagonistic effects on cytokine secretion although some modest modulations can be observed.

We monitored also the effect of LRAs on cytokine gene expression using the same donors for the same time points. Our results shown in Figure 3 confirmed secretion assays since bryostatin‐1 treatment rapidly increased expression of pro‐inflammatory cytokines and decreased IL‐10. Although CCL5 expression increased at 24 h, a longer treatment tends to cause a restoration (IL‐8, IL‐10, and TNF‐α) or a reduction (CCL2) of gene expression compared to the untreated cells. JQ1 modestly decreased CCL2 and IL‐8 without affecting other genes. In contrast to bryostatin‐1, romidepsin treatment induced a delayed response decreasing CCL2, IL‐10, and TNF‐α while increasing CCL5 and IL‐8 expression only at 24 h. As observed in our secretion data, combinations of LRAs have minor effect on gene expression except with bryostatin‐1 and romidepsin. Altogether, these results indicate that LRAs profoundly impact several cytokine basal secretion in macrophage mainly through transcriptional modulations.

### 3.3 Physiologic functions of MDMs are mildly affected by LRAs

**Figure 4:**
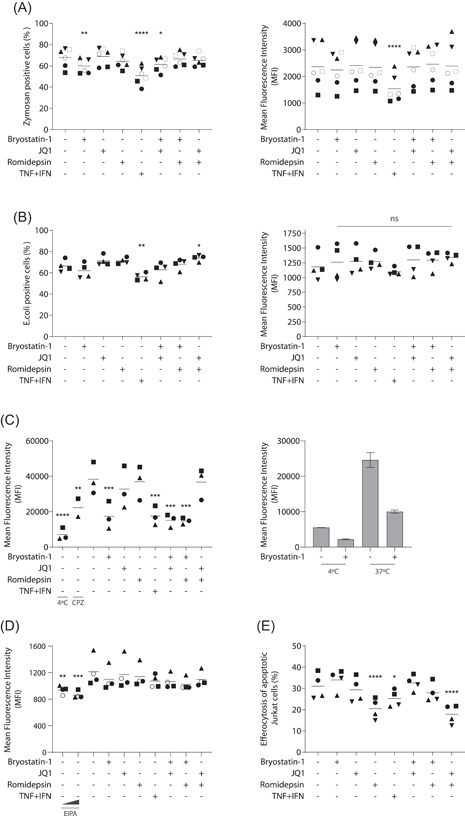
Effect of latency‐reversing agent (LRA) treatment on phagocytic and endocytic capacity of monocyte‐derived macrophage (MDMs). Cells were either left untreated or treated with LRAs for 24 h. Phagocytic capacity was quantified by the engulfment of pHrodo‐labeled zymosan (A) (*n* = 6) or *Escherichia coli* (B) (*n* = 4) particles. Percentages of positive cells and mean fluorescence intensities are represented in left and right panels, respectively. (C) Endocytosis (left panel, *n* = 3) or binding (right panel, *n* = 2) of transferrin was also monitored as well as (D) uptake of pHrodo‐labeled Dextran (*n* = 4). The mean fluorescence intensity of the cell population is represented. (E) Efferocytosis of apoptotic Jurkat cells by MDMs is shown (*n* = 4). Data are displayed as the percentage of MDMs positive for Apotracker green stained Jurkat cells. All experiments were performed by flow cytometry. One‐way ANOVA with Dunnett's multiple‐comparison test (*p ≤ .05. **p ≤ .01. ***p ≤ .005. ****p ≤ .001)

We then assessed whether the LRAs‐induced morphological, transcriptomic, and proteomic modulations of MDMs could influence primary immune capacities of human macrophages. Since phagocytosis represents one of the main immune functions of these cells, we first quantified the yeast and bacteria engulfment capacity using pHrodo‐labeled Zymosan (Figure 4A) and *E. coli* particles (Figure 4B), respectively. As depicted in Figure 4A, Zymosan phagocytosis was slightly reduced when cells were treated with bryostatin‐1 for 24 h. However, this effect was much smaller from what could be observed with the pro‐inflammatory like phenotype which is known to decrease phagocytosis uptake ^24^, ^25^ and was not potentiated by combination with any other LRAs. On the other hand, no significant LRAs‐induced alterations of *E. coli* phagocytosis could be observed.

Next, we investigated the modulatory effect of LRAs on different endocytic routes such as clathrin‐mediated uptake using labeled human transferrin (Figure 4C) and phase fluid uptake or macropinocytosis using pHrodo‐labeled dextran particles (Figure 4D). EIPA and chlorpromazine were used as negative controls to prevent phase fluid and receptor‐mediated endocytosis, respectively. Receptor‐mediated endocytosis was markedly impaired in cells treated with bryostatin‐1 and TNF + IFN, reaching similar levels when compared with the clathrin inhibitor chlorpromazine. Similar experiments performed at 4°C to prevent active endocytosis but not binding revealed that bryostatin‐1 treatment also resulted in a major reduction of the mean fluorescence intensity (MFI) suggesting a reduction of transferrin binding (Figure 4C). Indeed, the ratio of MFI measured at 37°C over 4°C was similar in bryostatin‐1‐treated and untreated cells, thus suggesting that receptor binding rather than endocytosis flux or clathrin‐mediated endocytic pathway impairment is the main mechanism responsible for the bryostatin‐1‐mediated diminution of transferrin endocytosis. In addition, dextran uptake was only slightly reduced by bryostatin‐1 and TNF + IFN treatments while other conditions, aside from the positive control EIPA, did not alter macropinocytosis (Figure 4D).

Finally, clearance of apoptotic cells by efferocytosis was estimated by incubation of LRAs‐treated and DID‐labeled MDMs with labeled apoptotic Jurkat cells (Figure 4E). Binding or engulfment of apoptotic Jurkat cells was inhibited by romidepsin treatment, used either alone or in combination with JQ1. Conversely its combination with bryostatin‐1 tends to rescue efferocytosis close to the level seen in untreated cells.

Overall, these results suggest that LRAs can impact specific phagocytic and endocytic functions of human primary macrophages.

### 3.4 MDMs treated with bryostatin‐1 do not express typical markers of classically activated macrophages

**Figure 5:**
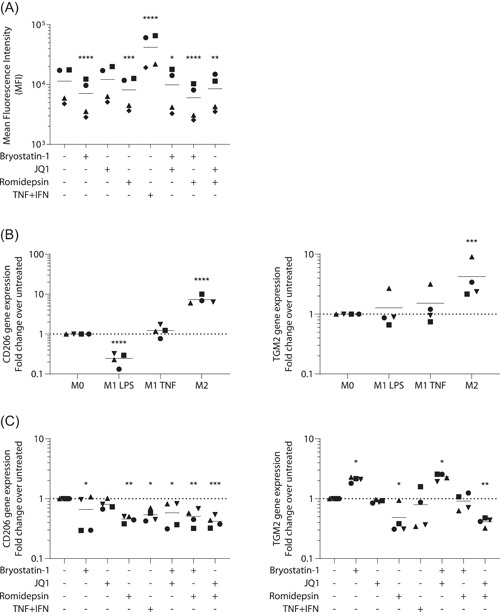
Latency‐reversing agents (LRAs) affect macrophage polarization markers. Monocyte‐derived macrophage (MDMs) were treated for 24 h with listed LRAs or polarization cytokines. (A) Mean fluorescence intensity of ICAM‐1 detection by flow cytometry (*n* = 4). Data were log transformed before statistical analysis. (B) Gene expression of CD206 (left panel) and TGM2 (right panel) was determined for unpolarized (M0) and MDMs treated with LPS + IFN (M1 LPS) TNF + IFN (M1 TNF) and IL‐4 (M2) cells or (C) LRA‐treated MDMs by qPCR (*n* = 4). One‐way ANOVA with Dunnett's multiple‐comparison test (*p ≤ .05. **p ≤ .01. ***p ≤ .005. ****p ≤ .001)

Because the bryostatin‐1‐induced modulation of macrophage physiology and functions is remarkably similar to the classically activated macrophage phenotype, we sought to determine whether LRAs could impact macrophage polarization. Since this process is highly dependent on cellular microenvironment,^26^ we characterized macrophage markers expressed in our own in vitro model. TGM2 and CD206 are considered as markers of alternatively activated macrophages ^27^ while Indoleamine 2,3‐dioxygenase‐1 (IDO‐1) and ICAM‐1 are upregulated in classically activated macrophages.^28^, ^29^, ^30^ Based on morphological alterations observed in Figure 1D, we first quantified surface expression of the adhesion molecule ICAM‐1 by flow cytometry. While the TNF and IFN combination was indeed associated with an upregulation of ICAM‐1 expression, treatment with bryostatin‐1 and romidepsin induced a trend towards its downregulation (Figure 5A).

Since cellular clustering was only observed in bryostatin‐1‐treated MDMs, our results suggest that ICAM‐1 downregulation is not involved in this alteration. We then compared expression of the polarization markers described above by qPCR on unpolarized, alternatively (IL‐4) and classically activated macrophages (LPS or TNF and IFNγ). Results depicted in Figure 5B confirmed that CD206 and TGM2 are upregulated in alternatively macrophages as expected (about 9‐fold and 4‐fold, respectively). Moreover, CD206 expression is downregulated by four‐fold when IFNγ is combined with LPS but not with TNF, therefore suggesting that typical polarization marker can be differently modulated by the microenvironment. On the other hand, IDO‐1 expression was, as expected, not found in unpolarized and alternatively activated macrophages and readily detected in the M1‐like phenotypes (data not shown).

Based on these observations, we finally quantified expression of these genes on MDMs treated for 24 h with the studied LRAs (Figure 5C). While IDO‐1 expression was not detected by any treatments except for the TNF and IFN combination (data not shown), CD206 and TGM2 were efficiently modulated by LRAs. Surprisingly, romidepsin induced a two‐fold decrease in both of these genes while bryostatin‐1 upregulated TGM2 (two‐fold) and decreased CD206 with high donor‐to‐donor variations. When combined together, romidepsin and bryostatin‐1 appear to compensate each other's impact on TGM2 gene expression, resulting in a baseline level (Figure 5C). These results thus suggest that bryostatin‐1 and romidepsin treatments modulate typical M1 and M2 activation markers but do not seem to mediate a defined polarization status.

### 3.5 J‐Lat reactivation process is downregulated by conditioned medium from LRAs‐treated MDMs

**Figure 6:**
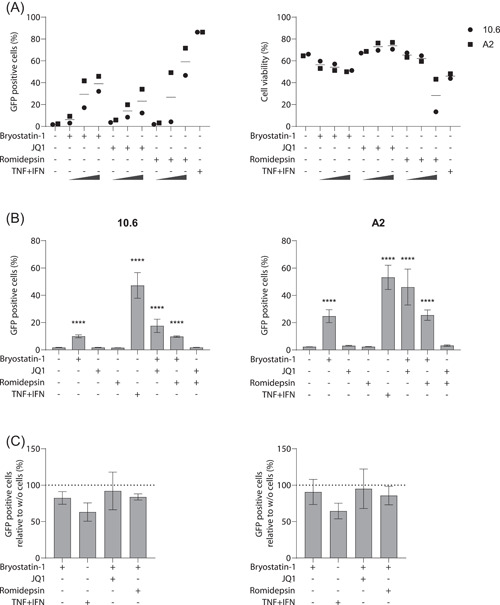
Reactivation of HIV‐1 in J‐Lat cells is not mediated by conditioned medium from latency‐reversing agent (LRA)‐treated monocyte‐derived macrophage (MDMs) (A) J‐Lat cells clone 10.6 or A2 were treated for 24 h with increasing concentrations of listed LRAs or (B,C) conditioned medium from MDMs. (A) Percentage of reactivation based on GFP expression (left panel) and cell viability by dye exclusion (right panel) are represented. (B) Conditioned media from LRA‐treated MDMs (24 h) of five donors were used to reactivate J‐Lat cells clone 10.6 (left panel) and A2 (right panel) and (C) compared to cell‐free LRA‐containing medium. Because JQ1 and romidepsin alone did not lead to substantial amount of reactivation, these conditions were not displayed. All experiments were performed by flow cytometry. One‐way ANOVA with Dunnett's multiple‐comparison test (****p ≤ .001)

Since LRAs are primarily studied to reverse HIV‐1 latency in CD4^+^ T cells, we assessed their reactivation potency on J‐Lat 10.6 and A2 latency cell line models.^31^ Cells were first treated for 24 h with increasing LRA concentrations (i.e., 1, 5, and 25 nM for romidepsin; 4, 20, and 100 nM for bryostatin‐1; and 100, 500, and 2500 nM for JQ1). The HIV‐1 LTR driven transcriptional activity was quantified based on GFP expression using flow cytometry (Figure 6A). Compared to untreated cells, bryostatin‐1 was the only LRA to achieve a moderate increase of GFP expression within both cell lines at the noncytotoxic concentrations used in our MDMs studies, while romidepsin and JQ1 were seemingly ineffective. As expected, reactivation with bryostatin‐1 was weaker than with the TNF and IFN combination. Moreover, with the exception of JQ1, the maximum LRAs concentration used while resulting in a slight increase of J‐Lat reactivation induced a substantial increase in toxicity, especially with romidepsin.

We next assessed whether conditioned medium from LRAs‐treated macrophages could influence HIV‐1 reactivation. To this end, J‐Lat cells were incubated for 24 h with conditioned medium from LRAs‐treated macrophages at the noncytotoxic doses (Figure 6B). We found a reactivation pattern similar to that obtained with LRAs used alone at similar concentrations. Since LRAs were not removed from the conditioned medium, we finally compared the reactivation pattern obtained with conditioned media to that obtained with cell‐free, LRAs‐containing media. As observed in Figure 6C, macrophages conditioned medium did not potentiate HIV‐1 reactivation in J‐Lat cells clone 10.6 (left panel) or A2 (right panel) with even a trend towards its downregulation in both clones. Our results thus suggest that the LRAs‐treated macrophage secretome may not favor HIV‐1 reactivation in CD4^+^ T cells.

## 4 DISCUSSION

Because the shock and kill strategy requires a potent immune capacity to purge HIV‐1, assessing the impact of LRAs on some physiological cell features thus appears critical. With the recent identification of an inducible viral reservoir in human macrophages despite prolonged combined antiretroviral therapy (cART)^10^ and given their central role in homeostasis, the possible impact of LRAs on such cell type needs to be addressed.

Hence, our work focused on monitoring the overall effect of LRAs on some physiologic features of uninfected human macrophages. Concentrations of LRAs tested in this study were chosen based on previous reports on CD4^+^ T cells and latency reporter cell lines to efficiently reverse latency while limiting cell toxicity. As for CD4^+^ T cells, our data revealed only minor modulations in cellular viability of human macrophages. However, while bryostatin‐1 induced resistance to apoptosis cell death in CD4^+^ T cells^32^ and did not present major toxic activity in clinical studies^33^, ^34^ or in a in vivo latency reversal trial,^35^ our work shows a minor apoptotic promotion in macrophages, similar to what is seen in CD8^+^ T cells studies.^17^ Nevertheless, in vivo studies used very low concentrations of bryostatin‐1 ranging in picomolars in the plasma, our apparent toxicity could therefore be attributed to higher drug concentrations combined with limited metabolization.

While global T‐cell activation appears unaffected by LRA treatments,^5^, ^6^ cytokine secretion and expression are often overlooked in reactivating studies in CD4^+^ T cells. Yet, some work seems to exclude cytokine secretion as a result of LRAs treatment in this cell type.^5^ In contrast, our data indicate a rapid activation of MDMs skewed toward a pro‐inflammatory response by bryostatin‐1 whereas JQ1 displayed an anti‐inflammatory effect. These observations were not surprising giving the well‐documented knowledge of PKC activation or Bromodomain and Extraterminal motif protein inhibitors (BETis) impact on cytokines secretion and echoed previous in vitro studies performed on monocytes and astrocytes.^21^, ^36^ While we did not observe major immunomodulation by romidepsin, we did, however, find a progressive decreased metabolic activity of MDMs treated by this HDACi which may be related to this family cell cycle progression arresting properties in human macrophages.^37^

Modulation of pro‐inflammatory cytokines may represent a double edge sword during latency reversal. Indeed, all cytokines assessed in this study are known to be upregulated by HIV‐1 infection and have an incidence on its pathogenesis. IL‐8 and CCL2 are known to increase blood‐brain barrier permeability as well as monocytes and neutrophils transmigration, fueling neuroinflammation and HIV‐1 propagation in this sanctuary.^38^, ^39^ In addition, they enhance HIV‐1 replication in MDMs.^40^, ^41^ Finally, their plasmatic levels are believed to correlate with chronic inflammation and poorer clinical outcome in cART‐treated patients.^42^, ^43^ Hence, their upregulation by bryostatin‐1 could have deleterious clinical impacts. On the other hand, boosting the immune system is often proposed to cope with HIV‐1 immune exhaustion to eradicate reactivated cells. As such, bryostatin‐induced MDM‐derived pro‐inflammatory cytokines could recruit immune cells and counterbalance its decreased “kill” features seen in CD8 T cells.^17^ Another advantage of pro‐inflammatory cytokine secretion would reside in their ability to reactivate latent cells. However, conditioned media from bryostatin‐1 or TNF + IFN‐treated macrophages had poor reactivation potential in the J‐Lat model, compared to LRA alone. These results could thus imply that the secretome from treated macrophages do not favor or may even reduce the reactivation of neighboring latently infected cells or may be the consequences of LRA uptake and metabolization by macrophages.

Because HIV‐1 infection impairs numerous macrophage functions such as phagocytosis, we assessed whether LRAs would impact uninfected cells.^44^ We observed only a minor trend toward reduction of the phagocytic capacity of *E. coli* and Zymosan particles following bryostatin‐1 treatment, a minor impact compared to the downregulation observed in fetal astrocytes.^22^ Moreover, in contrast to astrocytes, JQ1 had no impact on macrophage phagocytic capacity, therefore suggesting a cell‐type specific effect. On the other hand, romidepsin induces a significant inhibition of apoptotic cell clearance. This may be linked by downregulation of TGM2 expression, a receptor involved in efferocytosis following romidepsin treatments.^45^ An inability to remove apoptotic cells may contribute to secondary necrosis in which apoptotic vacuoles burst and release their toxic content, causing chronic inflammation and possible production of auto‐immune antibody responsible for various auto‐immune disorders.^46^ In addition to its global homeostatic functions, efferocytosis is also involved in clearance of numerous pathogens within infected cells such as *Mycobacterium tuberculosis*,^47^ a common co‐infection in people living with HIV. However, some pathogens including *Leishmania major* can exploit this process as Trojan horse to directly infect macrophages.^48^, ^49^, ^50^ Therefore, inhibition of efferocytosis by romidepsin may be of interest to boost immune responses but could worsen chronic inflammation and modulate specific co‐infections.

Macrophages constitute a highly plastic cell population, which displays a broad spectrum of polarization phenotypes that are tightly regulated by the cellular microenvironment.^51^ On both ends of this spectrum can be pictured two extreme cell status: the pro‐inflammatory or M1 and the anti‐inflammatory or wound healing M2 macrophages, both leading to a decrease in HIV‐1 infection.^52^ Because we and others have previously shown that romidepsin and bryostatin‐1 decrease de novo infection in macrophages and CD4^+^ T cells,^53^, ^54^, ^55^ we studied the impact of LRAs on their polarization status. Our data revealed striking similitudes between our M1‐like pro‐inflammatory control and bryostatin‐1‐treated macrophages ranging from morphology to secreted cytokines to endocytic and somewhat phagocytic behaviors.^24^, ^52^, ^56^ However, we found that romidepsin downregulated M2 expression markers while bryostatin‐1 upregulated TGM2 and downregulated CD206. Furthermore, these LRAs induced a decrease in ICAM‐1 surface expression and did not upregulate IDO‐1, both M1 markers. Because IDO‐1 expression is induced by both type I and II interferons, we could suggest that LRA treatments do not trigger such a response.^29^ Thus, LRA‐treated macrophages may harbor an intermediate polarization status which would require intensive transcriptomics and proteomics data to characterize.

With an insufficient potency to reactivate HIV‐1 when used alone, combinations of LRAs of different classes were shown to be more effective as our J‐Lat studies tend to confirm. However, these combinations did not produce dramatic modulations of macrophage physiologic features. Still, some modest fluctuations could be observed when LRAs modulate similar or opposite macrophages functions. This mild response could be explained by the weak impact of romidepsin and JQ1 on studied features, imposing bryostatin‐1 as the main effector. This would thus suggest that unlike our findings on astrocytes,^21^ JQ1 anti‐inflammatory features are unable to efficiently dampen bryostatin‐1‐induced pro‐inflammatory state in macrophages.

Overall, our study provides some insights on the effect of LRAs on some physiologic features of primary macrophages. We could identify bryostatin‐1 as a pro‐inflammatory agent in human macrophages. We also show that pro‐inflammatory macrophage secretome may decrease HIV‐1 reactivation of neighboring cells. Given that HIV‐1‐infected individuals are more prone to secondary viral and bacterial infections which modulate the overall immune response (i.e., normal, pro‐inflammatory, or immunosuppression), LRAs treatment could translate either into beneficial or detrimental effects. Additional in vitro and in vivo studies are warranted to more precisely investigate the various LRA‐mediated effects on numerous immune cell subtypes to define the pros and cons of the shock and kill strategy.

## CONFLICT OF INTERESTS

The authors declare no conflict of interest.

## AUTHOR CONTRIBUTIONS

Laurent Hany, Marc‐Olivier Turmel, Corinne Barat, and Michel Ouellet performed the research. Corinne Barat and Michel Ouellet designed the research study. Laurent Hany, Marc‐Olivier Turmel, Corinne Barat, and Michel Ouellet analyzed the data. Laurent Hany, Marc‐Olivier Turmel, Corinne Barat, Michel Ouellet, and Michel J. Tremblay wrote the paper. All authors have read and approved the final manuscript.

## Data Availability

The data that support the findings of this study are available from the corresponding author upon reasonable request.
